# Association between in-hospital nursing workload and early functional recovery after unilateral arthroscopic rotator cuff repair: a retrospective cohort study

**DOI:** 10.3389/fphys.2026.1862390

**Published:** 2026-09-24

**Authors:** Jiajie Yan, Lan Li, Yali Xu, Cuiwen Zhang, Qiang Teng, Jiamin Zhang, Xiaoxin Zhang, Meili Liu, Xiaofei Zheng, Haobo Zhong, Qiuyan Chen

**Affiliations:** 1 Department of Sports Medicine, The First Affiliated Hospital of Jinan University, Guangzhou, China; 2 Department of Nursing, The First Affiliated Hospital of Jinan University, Guangzhou, China; 3 Department of Orthopaedics, Xinfeng County People’s Hospital, Ganzhou, China; 4 Department of Sports Medicine, The First Affiliated Hospital of Jinan University, Guangzhou Key Laboratory of Precision Orthopedics and Regenerative Medicine, Guangdong Key Laboratory of Speed Ability Research, Guangzhou, Guangdong, China; 5 Department of Orthopaedic, Huizhou First Hospital, Guangdong Medical University, Huizhou, Guangdong, China

**Keywords:** arthroscopic rotator cuff repair, ASES score, early functional recovery, nursing workload, retrospective cohort study

## Abstract

**Background:**

Early functional recovery after arthroscopic rotator cuff repair (ARCR) varies substantially among patients. Although surgical characteristics and rehabilitation strategies have been widely investigated, few studies have examined patient-level early postoperative nursing workload as an integrated indicator of care complexity after ARCR.

**Methods:**

This single-center retrospective cohort study included 202 patients who underwent unilateral ARCR between January 2022 and December 2024. Patient-level nursing workload within the first 48 hours postoperatively (NWI-48h) was quantified using structured nursing records. The primary outcome was the American Shoulder and Elbow Surgeons (ASES) score at 3 months. Multivariable linear regression and logistic regression analyses were performed with progressive adjustment for potential confounders.

**Results:**

Higher NWI-48h was associated with lower ASES scores at 3 months. In the fully adjusted model, each 1-SD increase in NWI-48h was associated with a 4.21-point decrease in ASES score (95% CI, −7.04 to −1.38; P = 0.004). Patients in the high-workload group had lower 3-month ASES scores than those in the low-workload group, with a graded association across workload tertiles. For unplanned healthcare visits or hospital readmissions, adjusted estimates were imprecise, with wide confidence intervals, and should be regarded as hypothesis-generating.

**Conclusion:**

Higher early postoperative patient-level nursing workload was associated with poorer short-term functional recovery after unilateral ARCR. NWI-48h may serve as a candidate indicator of early postoperative care complexity that warrants prospective validation before clinical application.

## 1 Introduction

Rotator cuff tears are a common cause of shoulder pain and functional impairment, and arthroscopic rotator cuff repair (ARCR) is a commonly used surgical treatment (Mancini et al., 2023; Bedi et al., 2024). Despite continuous improvements in surgical techniques and perioperative management, early postoperative functional recovery remains highly variable across patients (Sahoo et al., 2026). Previous studies have primarily focused on tear characteristics, surgical techniques, and rehabilitation protocols, yet these factors do not fully explain the observed heterogeneity in recovery outcomes (Watson et al., 2018; Sahoo et al., 2026; Sussiek et al., 2026).

Increasing evidence suggests that the early postoperative period plays a critical role in determining long-term functional recovery (Choi et al., 2025). Effective pain control, early mobilization, and prevention of complications have been identified as key contributors to improved outcomes within the first three months after ARCR (Nakamura et al., 2018; Hu et al., 2023; Malavolta et al., 2023). However, most studies evaluate these factors separately, and few have attempted to capture the overall intensity of postoperative care using routinely available clinical data (Fagerstrom et al., 2018; Ross et al., 2023).

An important limitation in current research is the lack of objective and quantifiable indicators reflecting patient-level nursing care intensity during hospitalization. Nursing workload derived from structured nursing records may serve as a practical proxy for early postoperative care intensity rather than care quality. Several established nursing workload instruments, such as the Nursing Activities Score and TISS-28, were originally developed primarily for intensive care settings, whereas their applicability to patient-level workload assessment in routine orthopedic wards remains less established (Miranda et al., 1996; Miranda et al., 2003). Therefore, extending the concept of nursing workload measurement to patient-level assessment in orthopedic wards should be considered exploratory.

Conceptually, NWI-48h was not intended to capture tendon healing biology directly. Rather, high workload may reflect a pathway in which larger tears, longer operative durations, higher early postoperative pain, complications, and barriers to early rehabilitation increase nursing care needs; these same early postoperative burdens may be associated with delayed short-term functional recovery. Thus, NWI-48h is best interpreted as an integrated marker of early care complexity, not as a causal measure of nursing performance.

Therefore, this exploratory study constructed a Nursing Workload Index within the first 48 hours postoperatively (NWI-48h) based on routinely collected nursing information system data. Using a retrospective cohort design, we aimed to examine the association between early postoperative nursing workload and short-term functional recovery at 3 months after unilateral ARCR, while accounting for surgical complexity, patient characteristics, and early postoperative clinical factors.

## 2 Methods

### 2.1 Study design

This was a single-center retrospective cohort study including consecutive adult patients who underwent unilateral ARCR between January 2022 and December 2024. The date of surgery was defined as the index date. The exposure window was restricted to the first 48 hours after surgery, and patients were followed up to 3 months postoperatively (± 2 weeks). Given the retrospective design and the use of de-identified data, the requirement for informed consent was waived.

### 2.2 Inclusion and exclusion criteria

#### 2.2.1 Inclusion criteria

Patients were included if they met the following criteria: (1) aged ≥18 years; (2) diagnosed with a rotator cuff tear confirmed by imaging and intraoperative findings; (3) underwent primary unilateral arthroscopic rotator cuff repair (ARCR); (4) had complete structured nursing documentation within the first 48 hours postoperatively; (5) had available follow-up data at 3 months after surgery.

#### 2.2.2 Exclusion criteria

Patients were excluded if they met any of the following criteria: (1) underwent bilateral rotator cuff repair; (2) underwent revision surgery; (3) had concomitant major shoulder procedures (e.g., fracture fixation or shoulder arthroplasty); (4) had severe preexisting shoulder dysfunction unrelated to the index rotator cuff tear; (5) had incomplete clinical data or missing outcome measures.

### 2.3 Exposure assessment

The Nursing Workload Index (NWI-48h) was derived from structured nursing records extracted from the hospital information system. The index comprised three components: (1) frequency of nursing assessments and nursing rounds, (2) frequency of pain assessments, and (3) number of documented nursing interventions (e.g., injections, dressing changes, catheter care). Each component was standardized as a z score based on its distribution in the analytic cohort, and the composite index was calculated as the mean of the three standardized values. Given the lack of a validated weighting scheme for orthopedic ward patients after ARCR, equal weighting was applied. Accordingly, NWI-48h should be regarded as an exploratory, sample-dependent index that requires recalibration and validation before prospective application. NWI-48h was analyzed as both a continuous variable (per 1-SD increase) and a categorical variable (low, middle, high).

Operational definitions and counting rules for the three NWI-48h components are provided in **Supplementary Table 1**. Briefly, nursing assessments and nursing rounds were counted as distinct time-stamped nursing documentation entries; pain assessments included scheduled and as-needed structured pain-score documentation; and nursing interventions were counted as documented nurse-delivered interventions within the first 48 hours after surgery.

### 2.4 Outcome measures

The primary outcome was the American Shoulder and Elbow Surgeons (ASES) score at 3 months postoperatively (Patel et al., 2024). The secondary outcome was unplanned healthcare visits or hospital readmissions within 3 months. Covariates included demographic variables, comorbidities, surgical characteristics (e.g., tear size, operative duration), and early postoperative indicators such as pain scores, length of hospital stay, and complications (Zhao et al., 2021). The 3-month time point was selected *a priori* to capture early functional recovery during the inflammatory and early rehabilitation phase after ARCR; therefore, all conclusions were limited to short-term recovery.

### 2.5 Statistical analysis

Baseline characteristics were compared across NWI tertiles. Continuous variables were analyzed using one-way analysis of variance or the Kruskal–Wallis test, as appropriate, and are presented as mean (standard deviation). Categorical variables were compared using the chi-square test or Fisher’s exact test and are presented as counts (percentages). Multivariable linear regression models were used to assess the association between NWI-48h and ASES scores, while logistic regression was applied for binary outcomes. Three models with progressive adjustment were constructed. Subgroup analyses were performed according to age (<65 vs ≥65 years), and interaction terms were tested. No *a priori* sample-size calculation was performed because the sample comprised all eligible patients with complete exposure and outcome data during the study period; precision was therefore evaluated using 95% confidence intervals. Statistical analyses were performed using R version 4.3.1 (R Foundation for Statistical Computing, Vienna, Austria). A two-sided P value <0.05 was considered statistically significant.

## 3 Results

### 3.1 Baseline characteristics

As shown in **Table 1**, higher NWI-48h tertiles were characterized by greater surgical complexity and early postoperative clinical burden. Baseline imbalance was substantial: the low workload group had no large or massive tears, whereas 38.2% and 60.3% of patients in the high workload group had large and massive tears, respectively. Operative duration increased from 72.0 minutes in the low workload group to 116.2 minutes in the high workload group, mean postoperative pain scores at 48 hours increased from 3.2 to 6.2, and length of hospital stay increased from 2.9 to 5.8 days. These baseline patterns show that NWI-48h was closely associated with markers of surgical and early postoperative clinical complexity.

**Table 1:** Baseline and perioperative characteristics across NWI tertiles.

Characteristics	Low (n = 68)	Middle (n = 66)	High (n = 68)	P value
Age, years, mean (SD)	53.8 (10.9)	55.4 (10.7)	57.6 (11.9)	0.2
Sex, n (%)				0.6
Female	27 (39.7%)	32 (48.5%)	29 (42.6%)	
Male	41 (60.3%)	34 (51.5%)	39 (57.4%)	
BMI, kg/m², mean (SD)	22.0 (2.2)	22.1 (2.3)	22.4 (2.3)	0.6
Smoking, n (%)	21 (30.9%)	15 (22.7%)	21 (30.9%)	0.5
Diabetes mellitus, n (%)	2 (2.9%)	1 (1.5%)	10 (14.7%)	0.003
Tear size, n (%)				<0.001
Small	39 (57.4%)	1 (1.5%)	0 (0.0%)	
Medium	29 (42.6%)	29 (43.9%)	1 (1.5%)	
Large	0 (0.0%)	36 (54.5%)	26 (38.2%)	
Massive	0 (0.0%)	0 (0.0%)	41 (60.3%)	
Multiple-tendon involvement, n (%)	10 (14.7%)	14 (21.2%)	19 (27.9%)	0.2
Operative duration, min, mean (SD)	72.0 (13.5)	95.9 (13.8)	116.2 (13.6)	<0.001
Postoperative pain score at 48 h, mean (SD)	3.2 (0.9)	4.7 (0.9)	6.2 (1.0)	<0.001
Hospital length of stay, days, mean (SD)	2.9 (0.8)	4.3 (1.1)	5.8 (0.9)	<0.001
Any postoperative complication, n (%)	3 (4.4%)	4 (6.1%)	7 (10.3%)	0.4

### 3.2 Functional outcomes

ASES scores at 3 months decreased across increasing NWI tertiles (**Figure 1**). In multivariable linear regression analysis (**Table 2**), NWI-48h was associated with lower ASES scores (β = −4.21; 95% CI, −7.04 to −1.38; P = 0.004). When NWI-48h was analyzed categorically, both middle and high workload groups showed lower ASES scores compared with the low workload group, with a graded association across workload tertiles. The fully adjusted continuous estimate (-4.21 points) and high-versus-low tertile estimate (-6.43 points) were below reported minimal clinically important difference values for ASES after ARCR (approximately 13–17 points), suggesting that the observed association may be statistically detectable but may not reach typical patient-level thresholds for clinical importance (Mukadam et al., 2024).

**Figure 1:**
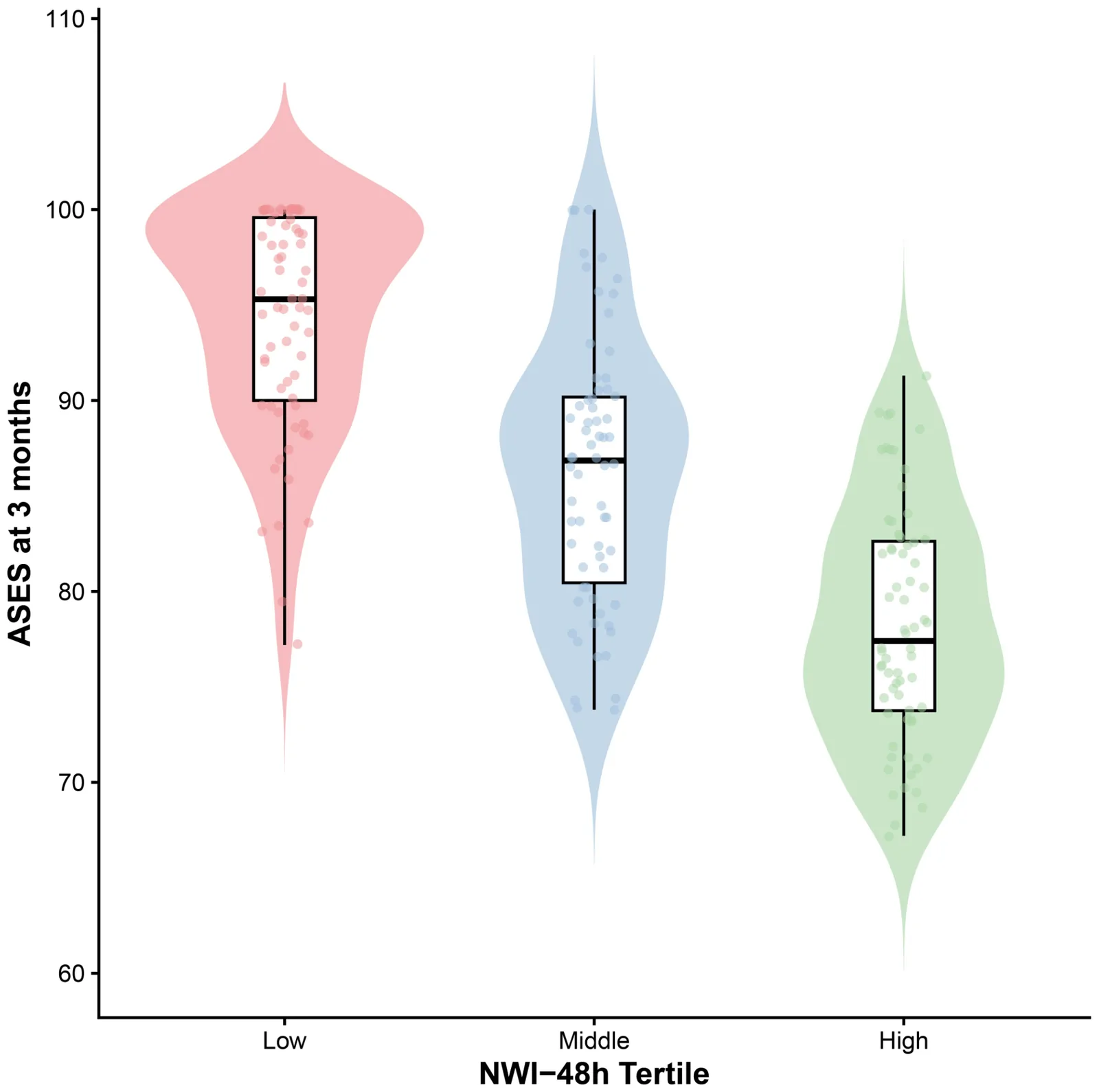
Distribution of ASES scores at 3 months across NWI-48h tertiles.

**Table 2:** Multivariable linear regression analysis for ASES scores at 3 months.

Model	Exposure	β (95% CI)	P value
Model 1	NWI (per 1 SD)	-6.68 (-7.50, -5.86)	<0.001
Model 2	NWI (per 1 SD)	-5.32 (-7.37, -3.27)	<0.001
Model 3	NWI (per 1 SD)	-4.21 (-7.04, -1.38)	0.004
Model 1	NWI tertile: High vs Low	-15.58 (-17.69, -13.48)	<0.001
Model 2	NWI tertile: High vs Low	-8.64 (-12.96, -4.32)	<0.001
Model 3	NWI tertile: High vs Low	-6.43 (-11.02, -1.84)	0.006
Model 1	NWI tertile: Middle vs Low	-7.98 (-10.08, -5.87)	<0.001
Model 2	NWI tertile: Middle vs Low	-4.16 (-7.27, -1.04)	0.009
Model 3	NWI tertile: Middle vs Low	-3.17 (-6.33, -0.01)	0.050

### 3.3 Secondary outcomes

Higher NWI-48h was associated with greater odds of unplanned healthcare visits or hospital readmissions in Model 1 (**Table 3**). After multivariable adjustment, however, the confidence intervals widened substantially. In Model 3, the high-versus-low workload comparison yielded an OR of 20.791 (95% CI, 2.171–268.346), indicating marked imprecision. Given the small number of events, these secondary-outcome estimates should be considered exploratory and hypothesis-generating.

**Table 3:** Multivariable logistic regression analysis for unplanned healthcare visits or hospital readmissions within 3 months.

Model	Exposure	OR (95% CI)	P value
Model 1	NWI (per 1 SD)	2.582 (1.746, 3.977)	<0.001
Model 2	NWI (per 1 SD)	1.903 (0.771, 4.828)	0.167
Model 3	NWI (per 1 SD)	1.359 (0.378, 4.93)	0.638
Model 1	NWI tertile: High vs Low	12.907 (4.614, 46.319)	<0.001
Model 2	NWI tertile: High vs Low	20.273 (2.47, 233.502)	0.008
Model 3	NWI tertile: High vs Low	20.791 (2.171, 268.346)	0.012
Model 1	NWI tertile: Middle vs Low	3.479 (1.13, 13.082)	0.041
Model 2	NWI tertile: Middle vs Low	7.071 (1.298, 60.136)	0.038
Model 3	NWI tertile: Middle vs Low	7 (1.247, 59.39)	0.041

4. Subgroup analysis.

In subgroup analyses (**Figure 2**), the negative association between NWI-48h and ASES scores was consistent across age groups, and no statistically significant interaction with age group was detected. For secondary outcomes, the direction of association remained similar, although estimates were less precise, particularly in older patients. Additional subgroup analyses by tear size or diabetes status were not considered stable in the present dataset because of extreme between-tertile imbalance and small numbers in some strata; these analyses should be treated as exploratory in future studies with larger samples.

**Figure 2:**
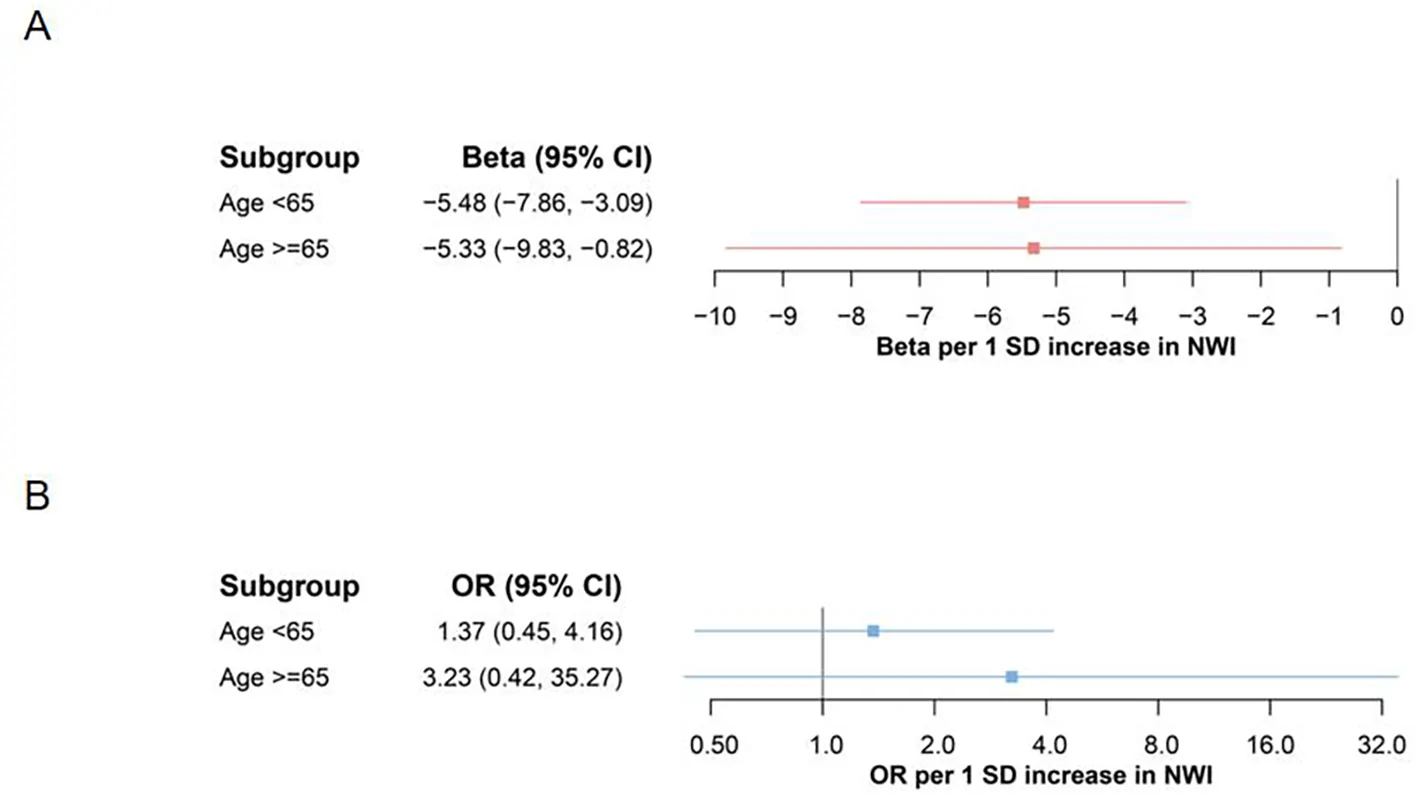
Subgroup analysis of the association between NWI-48h and outcomes stratified by age. **(A)** Association between NWI-48h (per 1-SD increase) and 3-month ASES scores stratified by age (<65 vs ≥65 years). **(B)** Association between NWI-48h (per 1-SD increase) and unplanned healthcare visits or hospital readmissions within 3 months stratified by age (<65 vs ≥65 years).

## 4 Discussion

This study identified three main findings. First, higher early postoperative nursing workload was associated with poorer functional recovery at 3 months after unilateral ARCR. Second, this association persisted after adjustment for demographic characteristics and surgical complexity, although the magnitude was below commonly reported MCID thresholds for ASES after ARCR. Third, further adjustment for postoperative factors attenuated the effect size, suggesting that part of the association may be related to early postoperative clinical status rather than an independent causal effect of nursing workload.

These findings align with previous studies emphasizing the importance of early postoperative management in functional recovery (Hu et al., 2023). Higher nursing workload may reflect greater postoperative burden, including higher postoperative pain, more intensive wound or drain care, and barriers to early rehabilitation. Importantly, NWI-48h should not be interpreted as a direct measure of tendon healing biology or nursing care quality. Rather, it may summarize a pathway in which surgical complexity and early postoperative symptoms increase nursing care needs, and these same early burdens are associated with delayed short-term functional recovery. Together, these findings support the notion that early nursing care intensity may serve as an indicator of recovery complexity rather than a direct causal factor.

The main contribution of this study is the construction of a quantifiable indicator of nursing workload based on routinely collected clinical data. Established workload instruments such as the Nursing Activities Score and TISS-28 were primarily designed for intensive care settings and workload/resource measurement rather than orthopedic ward recovery after ARCR (Miranda et al., 1996; Miranda et al., 2003).

NWI-48h was therefore constructed as a pragmatic patient-level measure tailored to routine ward documentation. Nevertheless, it remains exploratory. Future studies should compare NWI-48h with established prognostic factors, evaluate whether it improves model performance beyond tear size, operative duration, and pain trajectory, and assess complementary patient-reported outcomes such as PROMIS or SANE alongside ASES.

From a clinical perspective, NWI-48h may be useful only if it is available early enough to prompt action. A high NWI-48h in the first 48 hours could help identify patients who may benefit from closer pain reassessment, earlier rehabilitation coordination, reinforced discharge education, or more structured follow-up. These applications remain hypotheses and should be tested prospectively before implementation.

Several limitations should be considered. First, as a retrospective study, residual confounding and reverse causality cannot be excluded. Second, NWI-48h has not undergone external validation, formal content-validity assessment, inter-rater reliability testing, or comparison with established workload instruments; therefore, its construct validity remains uncertain. Third, NWI-48h was strongly associated with tear size, operative duration, postoperative pain score at 48 hours, and hospital length of stay, raising the possibility that it is a redundant summary of established prognostic factors rather than an independent predictor. Fourth, outcome-driven documentation may introduce information bias: patients with more severe postoperative symptoms may receive more assessments and nursing interventions, thereby increasing the measured exposure in the same patients who are more likely to have poorer recovery. Fifth, the complete-case design may introduce selection bias if missingness was related to exposure or outcome. Finally, only a small number of unplanned healthcare visits or hospital readmissions occurred, resulting in unstable secondary-outcome odds ratios with wide confidence intervals. These estimates are therefore hypothesis-generating rather than confirmatory and require validation in larger cohorts with more outcome events. Future multicenter studies with larger cohorts, prospective calibration, component-level validation, and incremental predictive analyses are needed to confirm these findings.

## 5 Conclusion

In this retrospective cohort, higher NWI-48h was associated with poorer short-term functional recovery after unilateral ARCR. NWI-48h should be considered a candidate indicator of early postoperative care complexity. Because the observed ASES effect was below commonly reported MCID thresholds and the index remains unvalidated, prospective validation against established prognostic factors is required before clinical application.

## Data Availability

The original contributions presented in the study are included in the article/**Supplementary Material**, further inquiries can be directed to the corresponding author/s.
