# Supplementary material for: Association between in-hospital nursing workload and early functional recovery after unilateral arthroscopic rotator cuff repair: a retrospective cohort study
Source: Front Physiol. 2026 Sep 24;17:1862390. doi: 10.3389/fphys.2026.1862390 (PMC13645609; doi:10.3389/fphys.2026.1862390)
Supplement: Supplementary file 1 [file Table1.docx]

**Supplementary Table 1. Operational definitions and counting rules for NWI-48h.**

| **NWI-48h component** | **Operational definition, counting rule, and data source** |
| --- | --- |
| Nursing assessments and ward rounds | Time-stamped postoperative nursing assessment entries, including routine ward observations, vital-sign/condition checks, wound or limb circulation assessments, and documented ward-round-related nursing observations during the first 48 hours after surgery. Each distinct time-stamped nursing information system entry was counted once. Duplicate edits to the same entry were not counted as new events. |
| Pain assessments | Structured pain-score documentation during the first 48 hours after surgery, including scheduled assessments and additional assessments before or after analgesic intervention. Each distinct numerical or structured pain assessment in the nursing information system was counted once. Repeated documentation at the same time point was treated as a single event. |
| Nursing procedures | Documented nursing procedures and interventions requiring nursing execution, including injections or infusion management, dressing changes, drain or catheter care, immobilization/brace assistance, cryotherapy assistance, and procedure-related patient education. Each time-stamped procedure entry was counted once. Bundled procedure sets were counted according to the documented event record in the hospital information system. |
| Composite NWI-48h | Patient-level composite workload index in the first 48 postoperative hours. The three component counts were standardized as z-scores within the analytic cohort and averaged with equal weights. The resulting score is sample-dependent and should be recalibrated before prospective use. |
